# Synthesis and Antimicrobial Activity of New Heteroaryl(aryl) Thiazole Derivatives Molecular Docking Studies

**DOI:** 10.3390/antibiotics11101337

**Published:** 2022-09-30

**Authors:** Victor Kartsev, Athina Geronikaki, Alexander Zubenko, Anthi Petrou, Marija Ivanov, Jasmina Glamočlija, Marina Sokovic, Lyudmila Divaeva, Anatolii Morkovnik, Alexander Klimenko

**Affiliations:** 1 InterBioScreen, 119019 Moscow, Russia; 2 School of Health, Faculty of Pharmacy, Aristotle University, 54124 Thessaloniki, Greece; 3 North-Caucasian Zonal Research Veterinary Institute, 346406 Novocherkassk, Russia; 4 Mycological Laboratory, Department of Plant Physiology, Institute for Biological Research “Siniša Stanković”, National Institute of Republic of Serbia, University of Belgrade, 11060 Belgrade, Serbia; 5 Institute of Physical and Organic Chemistry, Southern Federal University, 344090 Rostov-on-Don, Russia

**Keywords:** antimicrobial, antibacterial, antifungal, heteroaryl (aryl) thiazole derivatives, docking

## Abstract

Herein, we report the design, synthesis, and evaluation of the antimicrobial activity of new heteroaryl (aryl) thiazole derivatives. The design was based on a molecular hybridization approach. The in vitro evaluation revealed that these compounds demonstrated moderate antibacterial activity. The best activity was achieved for compound **3**, with MIC and MBC in the range of 0.23–0.7 and 0.47–0.94 mg/mL, respectively. Three compounds (**2**, **3**, and **4**) were tested against three resistant strains, namely methicillin resistant *Staphylococcus aureus*, *P. aeruginosa*, and *E. coli*, which showed higher potential than the reference drug ampicillin. Antifungal activity of the compounds was better with MIC and MFC in the range of 0.06–0.47 and 0.11–0.94 mg/mL, respectively. The best activity was observed for compound **9**, with MIC at 0.06–0.23 mg/mL and MFC at 0.11–0.47 mg/mL. According to docking studies, the predicted inhibition of the *E. coli* MurB enzyme is a putative mechanism of the antibacterial activity of the compounds, while inhibition of 14a-lanosterol demethylase is probably the mechanism of their antifungal activity.

## 1. Introduction

**Figure 1:**
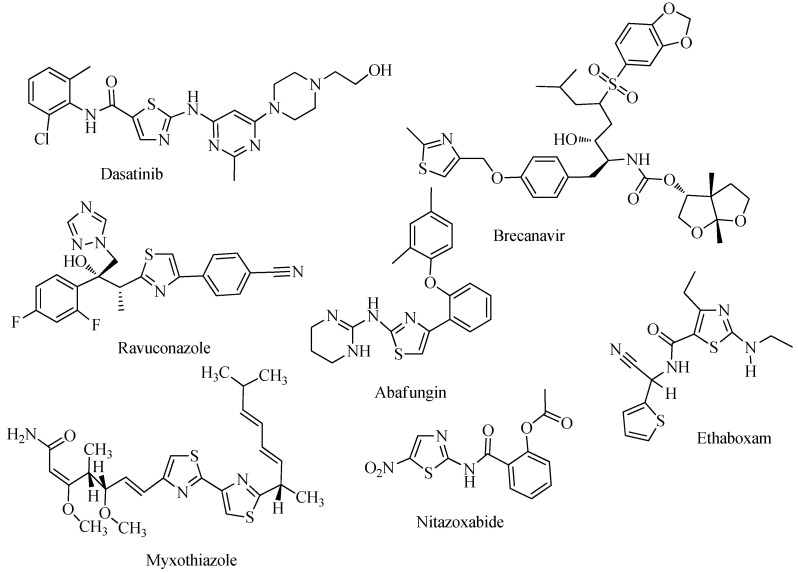
Thiazole-based approved drugs.

There is an increasing demand for the development of new antibacterial agents, due to global emerging resistance to conventional antibiotics. During the last several decades, a plethora of different thiazolidine based compounds have been studied to evaluate their pharmacological potential [1,2]. The synthesis of thiazole derivatives has attracted widespread attention due to their diverse biological activities, including antimicrobial [3,4,5,6,7,8,9], anti-inflammatory [10,11,12], analgesic [13,14], antitumor [15,16,17], antidiabetic [18], anti-HIV [19,20], COX/LOX inhibitory [21,22], antioxidant [23,24], antileishmanial [25,26], and many others [27,28,29,30]. There are many drugs with this scaffold such as antitumor (dasatinib, tiazofurin,); antiviral (brecanavir, ritonavir); anti-infectious (nitazoxanide) [31]; antibacterial agents, including sulfathiazole [32] and penicillins [33]; and antifungal agents, such as ravuconazole [34], myxothiazol [35], abafungin [36], and ethaboxam [37] (Figure 1).

Phtalazino derivatives are also mentioned as antimicrobial agents [38,39,40]. On the other hand, sulfonamides have attracted the interest of researchers due to their wide spectrum of biological activities, including dihydrofolate reductase (DHFR) inhibitors [41], antitumor [42,43], carbonic anhydrase inhibitors [44,45], anti-inflammatory [46], antiretroviral activity [47,48], antimicrobial [49,50], and others [51,52,53].

Sulfonamides are known as broad spectrum antimicrobial agents against *Gram-positive* and *Gram-negative* bacterial strains. These agents have low cost, low toxicity, and special activity against bacterial diseases. Sulfisoxazole, sulfamethizole, sulfamethoxazole, and sulfaphenazole are only some examples for the treatment of a diversity of bacterial infections.

Another example is the diuretic drug, chlorthalidone, which is used to treat hypertension or edema caused by heart failure, renal failure, hepatic cirrhosis, and estrogen therapy, as well as silver sulfadiazine, a topical sulfonamide antibiotic approved for the treatment of burns. Therefore, the design and development of hybrid molecules containing thiazolidinone phtalazine and thiazole cores, as well as sulfonamide groups, is a promising route in the search for novel antimicrobial agents. Molecular hybridization based on the amalgamation of two or more substitutions [54] is one of the new approaches in drug design. Hybridization is mainly aimed to improve the activity profile and to reduce undesired side effects [55].

Taking all of this information into account and based on our previous results [56,57,58], we designed and synthesized new derivatives incorporating thiazole, phtalazine moieties, and the sulfonamide group in the frame of one molecule.

## 2. Results and Discussion

### 2.1. Chemistry

**Scheme 1:**
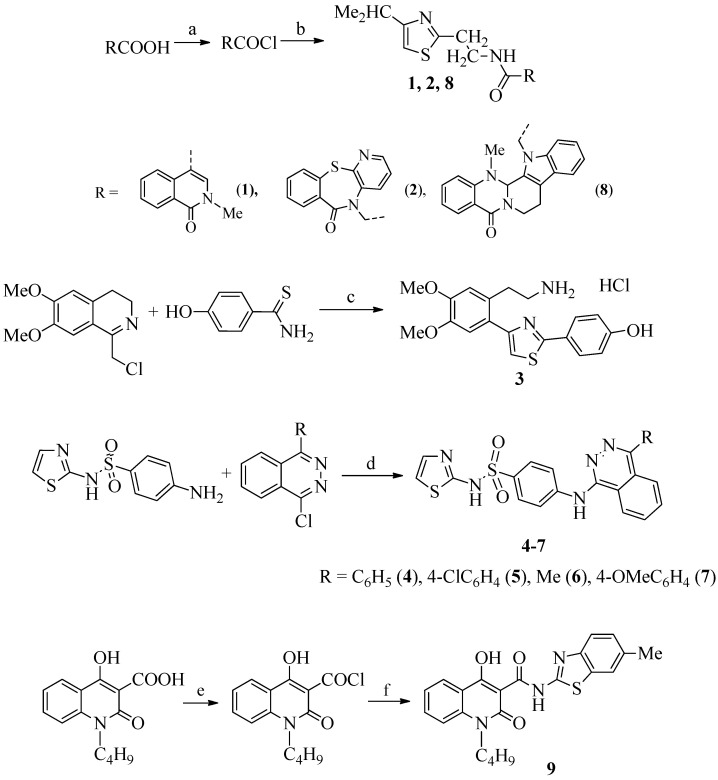
Synthesis of target compounds. Reagents and conditions: (a) RCOOH, SOCl_2_, CHCl_3_, DMF, reflux; (b) 2-(4-isopropylthiazol-2-yl)ethan-1-amine, NEt_3_, CHCl_3_, 0–2 °C, saturated aqueous solution of NaHCO_3_; (c) 1-(chloromethyl)-6,7-dimethoxy-3,4-dihydroisoquinoline, 4-hydroxybenzothioamide, i-PrOH, reflux, 2 h; (d) 1-chloro-4-R-phthalazine, 4-amino-N-(thiazol-2-yl)benzenesulfonamide, methyl cellosolve, reflux, 0.5 h, 95–100 °C, 1 h, 5% NH4OH; (e) 1-butyl-4-hydroxy-2-oxo-1,2-dihydroquinoline-3-carboxylic acid, SOCl_2_, CHCl_3_,DMF, reflux; (f) 6-methylbenzo[d]thiazol-2-amine, pyridine, DMF, 0 °C, 0.5 h, saturated aqueous solution of NaHCO_3_.

In this work, we have described the synthesis of three structural series of new thiazole derivatives and presented the results of a study of their antibacterial and antifungal activity against a number of bacterial and fungal pathogens. All the target compounds were synthesized via four routes, as outlined in Scheme 1.

Starting acids for the synthesis of compounds **1**, **2**, **8**, and **9**; 1-(chloromethyl)-6,7-dimethoxy-3,4-dihydroisoquinoline for the synthesis of thiazole **3**; and 1-chlorophthalazine derivatives for the preparation of compounds **4**–**7** were provided by InterBioScreen Ltd (Moscow, Russia).

Most of the compounds were synthesized according to the usual scheme, by acylation of the corresponding amines with acid chlorides. Compounds **1**, **2**, **8**, and **9** were obtained in these ways: acid chlorides 2-methyl-1-oxo-1,2-dihydroisoquinoline-4-carboxylic acid was used to synthesize compound **1**, 2-(6-oxobenzo[f]pyrido[2,3-b][1,4]thiazepin-5(6*H*)-yl)acetic acid for **2**, and 2-(14-methyl-5-oxo-7,8,13b,14-tetrahydroindolo[2′,3′:3,4]pyrido[2,1-b] quinazolin-13(5*H*)-yl)acetic acid for **8**, 1-butyl-4-hydroxy-2-oxo-1,2-dihydroquinoline-3-carboxylic acid for **9**; for these compounds, 2-(4-isopropylthiazol-2-yl)ethane-1-amine was used.

Compounds **4**–**7** were obtained by the reaction of nucleophilic substitution of the chlorine atom in 1-chloro-4-R-phthalazines. The best solvent for this reaction is methyl cellosolve, both in terms of yields and the purity of the reaction products. We especially note that the addition of ammonia for the conversion of product salts into bases should be carried out when the temperature of the reaction mixture is about 100 ^°^C, since neutralization at ordinary temperature takes a very long time and does not guarantee the complete conversion of salts into bases.

Compound **3** was synthesized using our rather unusual recyclization reaction of 1-chloromethyl-3,4-dihydroisoquinolines under the action of thioamides and thioureas.^.^In this article, the possibility of such recycling was confirmed by us, including using X-ray diffraction analysis and NMR spectroscopy (a one-proton singlet of the thiazole ring (H-5′) of all the compounds described in the article is observed in the region of 6.85–6.98 ppm).

In the present work, the spectra of the studied compounds are also characterized by the presence in their aromatic region of a one-proton singlet of the thiazole ring (H-5′) in the region of 6.01–7.04 ppm; in the case of compound **3**, this signal was detected at 7.44 ppm. 

In the ^1^H NMR spectra of compounds **1**, **2**, and **8** in deuterochloroform, the signals of the methyl groups of the isopropyl group are located in the upfield part of the spectra (1.21–1.28 ppm).

The amide proton NHCO in compounds **1** and **2** appears as a multiplet in the region 8.21–8.31, and in compound **8** it appears as a singlet at 8.08.

Compounds **4**–**7** contain two singlets: at 12.46–12.48 ppm of the sulfamide group NHSO_2_ and at 9.42–9.49 ppm of the NH group.

### 2.2. Biological Evaluation

#### 2.2.1. Antibacterial Activity

Synthesized compounds were tested for their antibacterial activity against a panel of six bacteria, using a microdilution method for the determination of minimal inhibitory and minimal bactericidal concentrations (MIC and MBC, respectively). The antibacterial activity of tested compounds was moderate to good, with MIC ranging from 0.17 to >3.75 mg/mL and MBC at 0.23–>3.75 mg/mL, as presented in Table 1. The order of activity can be presented as follows: **3** > **2** > **9** > **4** > **5** > **7** > **8** > **1** > **6**. The best activity was achieved for compound **3** with MIC and MBC at 0.23–0.70 mg/mL and 0.47–0.94 mg/mL, respectively. The most sensitive bacterium appeared to be *B. cereus*, whereas *E. coli* was the most resistant one.

Compound 4 exhibited the best activity among the compounds tested against *E. coli*, with MIC/MBC at 0.17/0.23 mg/mL, while compound **9** showed the same good activity against *B. cereus* and *S. Typhimurium*. Compounds **1** and **8** exhibited in vitro activity with MIC and MBC at 0.23/0.47 mg/mL against *E. cloacae*, compounds **3** and **5** against *E. coli*, while compound 3 also displayed good activity against *S. Typhimurium.* In general, these compounds showed moderate to low activity.

The study of structure–activity relationships revealed that the presence of 2-(3,4-dimethoxyphenyl)ethanamine as substituent at position 4 and phenol at position 2 of the thiazole ring (**3**) are beneficial for antibacterial activity. Among the group of compounds **1**, **2**, and **8**, the more favorable effect was observed in the case of 2-methylisoquinolin-1(2H)-one substituent connected via N-propylpropionamide with the thiazole ring (**2**). The presence of phenylphthalazine (**4**) as the substituent was positive in the case of compounds **4–7**. Introduction of 4-Cl substituent to phenylphthalazine (**5**) decreased a little activity, while the presence of the 4-OMe group decreased more activity. Finally, replacement of phenylphtalazine by 1-methylphtalazine (**6**) was detrimental for this group of compounds and in general for all tested compounds.

The evaluation of three of the most active compounds (**2**, **3**, **4**) against three resistant strains, namely methicillin-resistant *Staphylococcus aureus* (MRSA), *P. aeruginosa*, and *E. coli*, revealed that all compounds were found to be more potent against *MRSA* than ampicillin and streptomycin, which did not show a bactericidal effect. Compound **4** also seems to be more active than ampicillin against *P. aeruginosa* strain, but no compound was more active than the reference drug against *E. coli* (Table 2). The compounds also were evaluated for their ability to inhibit the biofilm formation. Unfortunately, no compound showed good activity.

#### 2.2.2. Antifungal Activity

Synthesized thiazolyl derivatives (**1–9**) were evaluated for their antifungal activity. For the determination of minimal inhibitory/fungicidal activity, the microdilution method was used [59]. 

All compounds showed good antifungal activity, and the results are presented in Table 3. The antifungal potency of synthesized compounds can be presented as follows: **8** > **9** > **1** > **3** > **5** > **2** > **4** > **6** > **7**. The best antifungal activity is achieved for compound **8**, with MIC at 0.08–0.23 mg/mL and minimum fungicidal concentration (MFC) at 0.11–0.47 mg/mL, whereas the lowest activity was observed for compound **7**, with MIC at 0.23–0.47 mg/mL and MFC at 0.47–0.94 mg/mL.

Thus, the sensitivity of the most resistant strain, *Aspergillus fumigatus*, toward the compounds tested is **3** > **5** = **8** = **9** > **1** = **2** > **4** = **6** = **7**, while for the most susceptible one, which is *Trichoderma viride*, the susceptibility can be presented as **9** > **8** > **1** = **3** > **5** = **6** > **2** = **4** > **7**. 

Ketoconazole showed antifungal potential at MIC 0.2–1.0 mg/mL and MFC 0.3–1.5 mg/mL, respectively, while bifonazole exhibited MIC at 0.1–0.2 and MFC at 0.2–0.25 mg/mL, respectively. Compounds **8** and **9** exhibited excellent activity, with MIC/MFC at 0.08/0.11 mg/mL, respectively, against *T. viride*, almost fourfold better than bifonazole and 29 times better than ketoconazole, as well as against *A. niger*, *A. versicolor*, *P. funiculosum*, and *P. cyclpoium var. verucosum*, with MIC/MFC at 0.11/0.23 mg/mL, respectievly. Good activity against *T. viride*, with MIC and MFC at 0.11 mg/mL and 0.23 mg/mL, respectively, was also displayed by compounds **1** and **3**, as well as by compounds **2**, **5**, and **6**, with MIC/MFC at 0.17/0.23 mg/mL, respectively. Compounds **3**, **5**, and **8** showed the same good activity against *A. niger*, with compound **8** also being potent against *P. cyclpoium var. verucosum.* It was observed that almost all compounds displayed better activity than ketoconazole against *T. viride*, with the exception of compounds **4** and **7**. In general, most of the compounds appeared to be more potent than ketoconazole against all fungi, except of *P.v.c.,* against which only three compounds (**1**, **8**, and **9**) were more active than ketoconazole.

The study of the structure–activity relationship revealed that the presence of 14-methyl-7,8,13b,14-tetrahydroindolo[2′,3′:3,4]pyrido[2,1-b]quinazolin-5(13H)-one as the substituent for compound (**8**), connected to position 2 of the thiazole ring via N-propylpropionamide, is beneficial for antifungal activity. The replacement for this substituent with the presence of 2-methylisoquinolin-1(2H)-one led to compound (**1**), with decreased activity. For the series of compounds (**1**, **2**, and **8**), the presence of 2-methylisoquinolin-1(2H)-one (2) was negative for antifungal activity. In the case of the substituted phthalazine-1-yl)amino)-N-(thiazol-2-yl)benzenesulfonamides, the most favorable structure for antifungal activity is the presence of a 4-chlorophenyl substituent in position 4 of the phtalazin ring in compound (**5**). Removal of 4-Cl-pnenyl substituent led to compound (**4**) having reduced activity. The least potent among all the compounds tested appeared to be compounds **6** and **7**, with methylphtalazine and 4-OMe phenylphtalazine substituents decreasing activity even more. The presence of 4-Me as well as 4-OMe-Ph substituents appeared to be detrimental to antifungal activity.

As a conclusion, the antifungal activity depends upon the substituents on the thiazole ring and, in the case of phthalazin-1-yl)amino)-N-(thiazol-2-yl)benzenesulfonamides, upon the substituents on the phtalazine ring. It should be mentioned that the antifungal activity of the synthesized compounds is much better than that of the antibacterial compounds.

### 2.3. Docking Studies

#### 2.3.1. Docking to Antibacterial Targets

In order to predict the possible mechanism of the activity of the tested compounds, docking studies were carried out on different targets. It is widely known that the most common mechanisms of activity of antibacterial agents are destroying the integrity of cell walls and cell membranes, inhibiting the expression of proteins, inhibiting the synthesis of nucleic acids, and affecting the energy metabolism of bacteria. In this direction, for the docking studies we used the enzymes responsible for these pathways, such as *E. coli* DNA gyrase, thymidylate kinase, *E. coli* primase, and *E. coli* MurA and *E. coli* MurB enzymes. 

Analyzing the docking studies scores, a low Free Energy of Binding represents a strong binding of a ligand to the enzyme. Taking this into account, the docking studies revealed that the Free Energy of Binding of all tested compounds to *E. coli* DNA gyrase, thymidylate kinase, and *E. coli* primase and *E. coli* MurA enzymes was higher than that of *E. coli* MurB (−7.02–−9.96 kcal/mol); therefore, it may be suggested that inhibition of *E. coli* MurB is probably the most suitable mechanism of action of the compounds where binding scores were consistent with biological activity (Table 4).

**Figure 2:**
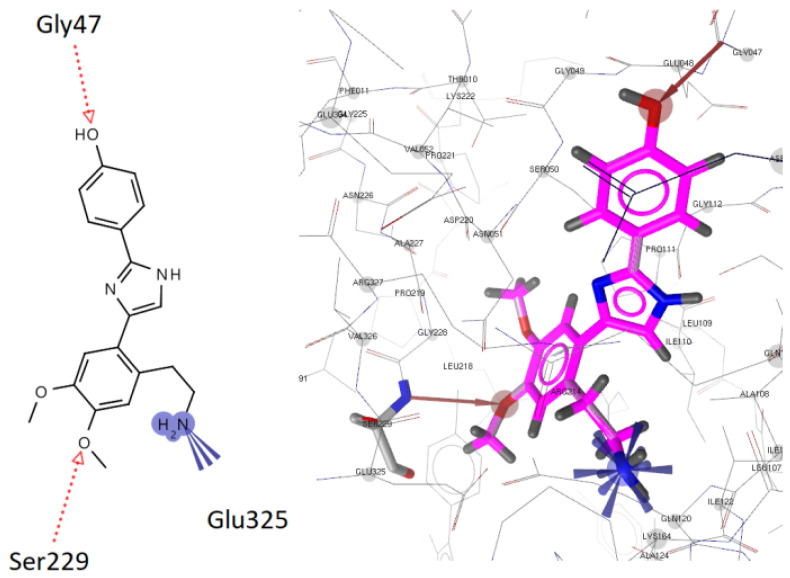
Docked conformation of the most active compound **3** in *E. coli* MurB. Red dotted arrows indicate H-bond, and blue lines indicate positive ionizable interactions.

**Figure 3:**
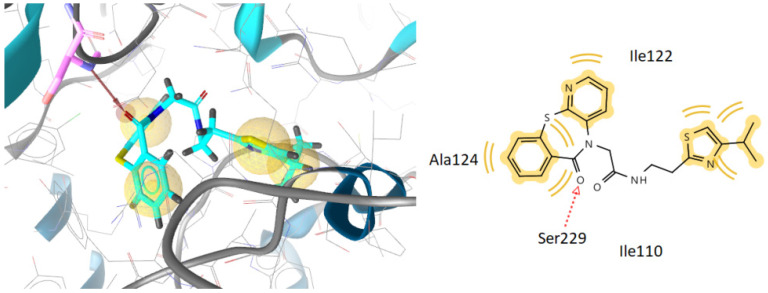
Docked conformation of the most active compound **2** in *E. coli* MurB. Red dotted arrows indicate H-bond, and yellow spheres indicate hydrophobic interactions.

The docking pose of the most active compound **3** in *E. coli* MurB enzyme showed two favorable hydrogen bond interactions. The first one was between the oxygen atom of -OH group of the compound and the hydrogen of the side chain of Gly47 (distance 2.25 Å), and the other hydrogen bond interaction was between the oxygen atom of the –OCH_3_ group of the compound and Ser229 residue (distance 2.73 Å). The NH_2_ group interacts with positive ionizable interaction with the residue Glu325, stabilizing the complex compound-enzyme and playing a vital role proving the high inhibitory action of compound **3**. Moreover, the hydrogen bond formation with the residue Ser229 is crucial for the inhibitory action of compound **3** as well as for compounds **2**, **4**, **8**, and **9**, because this residue takes part in the proton transfer at the second stage of peptidoglycan synthesis [60] (Figure 2 and Figure 3).

**Figure 4:**
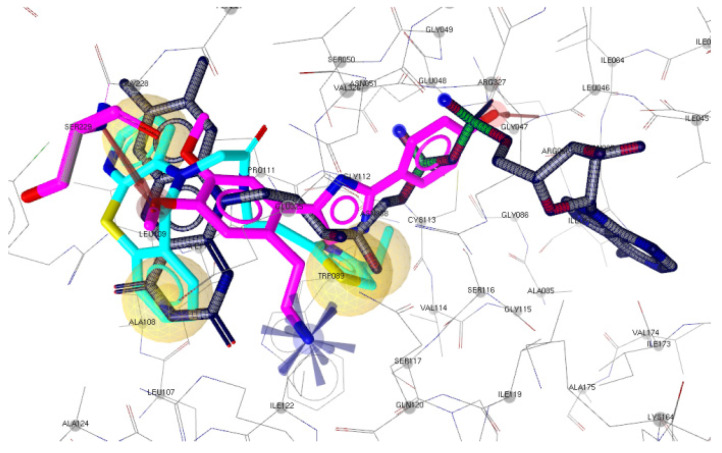
Superposition of compounds **3** (magenta), **2** (light blue), and FAD (gray) in *E. coli* MurB.

The second-most-active compound, compound **2**, also forms this hydrogen bond interaction with the residue Ser229, which explains its high inhibitory action (Figure 3). Detailed analysis of the docking pose of the two most active compounds showed that they bind MurB in a similar way as FAD, and they fit into the binding center of the enzyme, forming a hydrogen bond with the residue Ser229 (Figure 4).

#### 2.3.2. Docking to Antifungal Targets

All the synthesized compounds and the reference drug ketoconazole were docked to lanosterol 14α-demethylase of *C. albicans* and DNA topoisomerase IV (Table 5).

**Figure 5:**
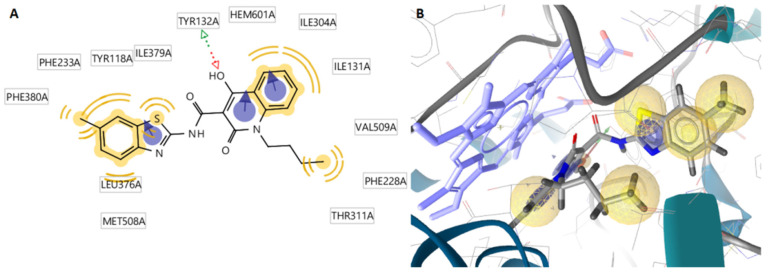
Docked conformation of the most active compound **9** in lanosterol 14α-demethylase of *C*. *albicans* (CYP51_ca_) (**A**,**B**). Red and green dotted arrows indicate H-bond, blue arrows indicate aromatic interactions, and yellow spheres indicate hydrophobic interactions.

**Figure 6:**
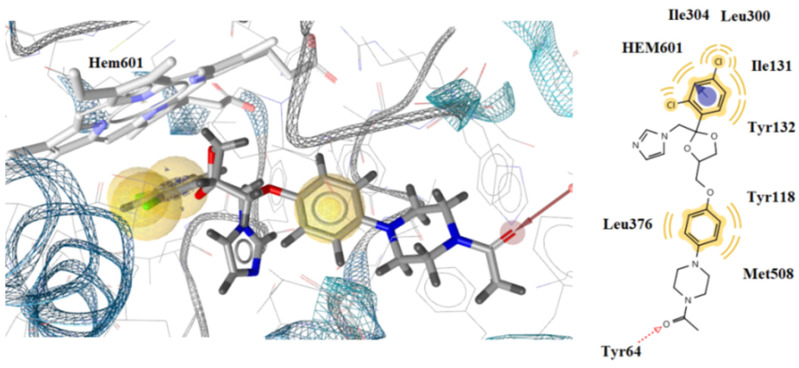
Docked conformation of ketoconazole in lanosterol 14α-demethylase of *C*. *albicans* (CYP51_ca_).

**Figure 7:**
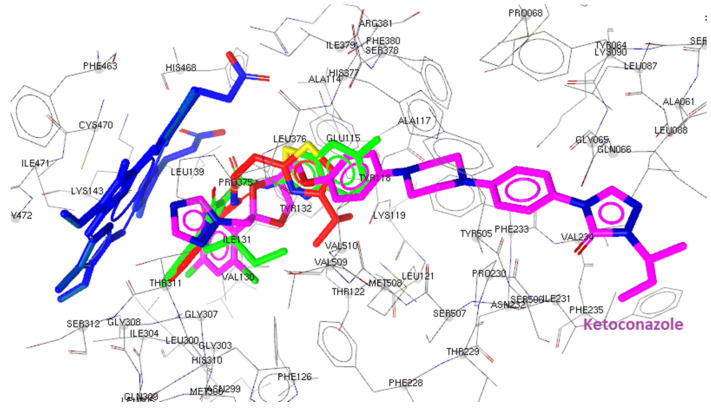
Superposition of compounds **9** (green), **1** (red), and ketoconazole (magenta) in lanosterol 14α-demethylase of C. albicans (CYP51_ca_). Heme group is illustrated in blue.

Docking results showed that the most active compound **9** binds the enzyme alongside the heme group, interacting with heme throughout its benzene ring, which forms aromatic and hydrophobic interactions. In addition, a hydrogen bond with Tyr132 residue and an -OH group of the compound are formed. Moreover, hydrophobic interactions between Ile304, ile131, Ile379, Ty188, Phe233, Phe380, Leu376, and Met508 and the compound were detected. Interaction with the heme group was also observed with the benzene ring of ketoconazole, which also forms aromatic interactions (Figure 5 and Figure 6). This property may account for why compound **9** has good antifungal activity. Superposition of compounds **9** and **1** and ketoconazole in the lanosterol 14α-demethylase of C. albicans (CYP51_ca_) shows this interaction with the heme group proving this hypothesis (Figure 7).

### 2.4. Drug-Likeness

**Figure 8:**
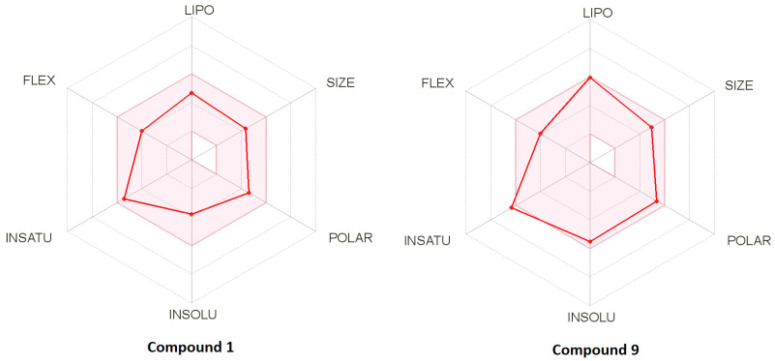
Bioavailability radar of compounds **1** and **9**. The pink area represents the optimal range for each property for oral bioavailability. Lipophilicity (LIPO): XLOGP3 between −0.7 and +5.0; molecular weight (SIZE): MW between 150 and 500 g/mol; polarity (POLAR) TPSA between 20 and 130 Å^2^; solubility (INSOLU): log S not higher than 6; saturation (INSATU): fraction of carbons in the sp3 hybridization not less than 0.25; flexibility (FLEX): no more than 9 rotatable bonds.

The bioavailability and drug-likeness scores of all compounds are shown in Table 6; according to prediction results, the bioavailability score of all compounds was about 0.55. Moreover, all compounds displayed good to excellent drug-likeness scores (−0.13–1.09). Figure 8 presents the bioavailability radar of some of the compounds. The best in the in silico predictions results was achieved for compounds **1** and **2**, with drug-likeness scores of 1.03 and 1.09, respectively, and with no violation of any rule.

## 3. Materials and Methods

### 3.1. General Information

^1^H NMR spectra of newly synthesized compounds were recorded on a spectrometer Bruker 400 (400 MHz); compound **6**—on spectrometer Bruker Fourier 300 (300 MHz) in DMSO-*d*_6_. Chemical shifts of nuclei ^1^H were measured relatively the residual signals of deuteron solvent (δ = 2.50 ppm; see Ref. (http://chem.ch.huji.ac.il/nmr/software/solvent.htmL (accessed on 1 August 2022)) and the literature cited therein). Coupling constants (*J*) are reported in Hz. Melting points were determined by using Fisher-Johns Melting Point Apparatus (Fisher Scientific) and are uncorrected. Elemental analysis was performed by the classical method of microanalysis. The reaction and purity of the obtained compounds were monitored by TLC (plates with Al_2_O_3_ III activity grade, eluent CHCl_3_, and development of TLC plates by exposition to iodine vapors in “iodine chamber”). The solvents were purified according to standard procedures. The starting compounds and compound **9** were provided by InterBioscreen Ltd. (Russia).

#### 3.1.1. General Procedure for the Synthesis of Compounds **1**, **2**, and **8**

A mixture of a corresponding acid (0.01 mol), SOCl_2_ (1.43 g, 0.87 mL, 0.012 mol), CHCl_3_ (20 mL), and DMF (0.05 mL) was refluxed until gas evolution stops and cooled, and the resulting solution of acid chloride was added dropwise at 0–2 °C to a solution of 2-(4-isopropylthiazol-2-yl)ethan-1-amine (1.7 g, 0.01 mol) and Et_3_N (2.02 g, 2.78 mL, 0.02 mol) in CHCl_3_ (15 mL). Then, NaHCO_3_ (9.5 g) and water (100 mL) were added and stirred, the organic layer was separated and dried with Na_2_SO_4_, and the solvent was distilled off in vacuum at 30–40 °C. The residue was purified by recrystallization from a suitable solvent.

*N-[2-(4-Isopropylthiazol-2-yl)ethyl]-2-methyl-1-oxo-1,2-dihydroisoquinoline-4-carboxamide* (**1**). The starting compounds were 2-methyl-1-oxo-1,2-dihydroisoquinoline-4-carboxylic acid and 2-(4-isopropylthiazol-2-yl)ethan-1-amine. Yield 2.63 g (74%), colorless crystals, m.p. 105–107 °C (CCl_4_). ^1^H NMR (400 MHz, DMSO-*d*_6_, δ, ppm): 1.28 (2s, 6H, 2Me), 3.24 (t, *J* 7.1, 2H), 3.56–3.67 (m, 6H, NMe, CHMe_2_), 6.93 (s, 1H, H-5′), 7.50 (d, *J* 7.4, 1H, H-5), 7.68 (d, *J* 7.5, 1H, H-8), 7.77 (s, 1H, H-3), 8.21–8.31 (m, 3H, H-6, H-7, NH). ^13^C NMR (126 MHz, DMSO-*d*_6_, δ, ppm): 22.68(4C), 33.03(4C), 11.83(2C), 125.22(1C), 127.42(5C), 156.13 (5C). Found (%): C, 64.51; H, 6.15; N, 11.56, S, 9.34. Calc. for C_19_H_21_N_3_O_2_S (%): C, 64.20; H, 5.95; N, 11.82, S, 9.02.

*N-[2-(4-Isopropylthiazol-2-yl)ethyl]-2-(6-oxobenzo[f]pyrido[2,3-b]*[1,4]*thiazepin-5(6H)-yl)acetamide* (**2**). The starting compounds were 2-(6-oxobenzo[f]pyrido[2,3-b][1,4]thiazepin-5(6*H*)-yl)acetic acid and 2-(4-isopropylthiazol-2-yl)ethan-1-amine. Yield 3.60 g (82%), colorless crystals, m.p. 141–143 °C (EtOAc). ^1^H NMR (400 MHz, DMSO-*d*_6_, δ, ppm): 1.26 (s, 3H, Me), 1.28 (s, 3H, Me), 2.96–3.07 (m, 5H, CHMe_2_), 3.13–3.18 (m, 2H, CH_2_CO), 6.93 (s, 1H, H-7), 7.10 (s, 1H, H-5′), 7.40–7.43 (m, 2H, H-4, H-8), 7.51–7.54 (m, 1H, H-3), 7.62–7.66 (m, 1H, H-2), 7.99 (dd, *J* 8.2, 1.6, 1H, H-9), 8.26–8.28 (m, 2H, H-5, NH). ^13^C NMR (126 MHz, DMSO-*d*_6_, δ, ppm): 168.07 (NHC=O), 167.86 (NC=O), 166.85, 162.86, 159.71, 146.65, 140.74, 137.25, 136.18, 133.92, 132.12, 132.10, 131.84, 129.46, 125.11, 115.02, 111.83, 54.67, 39.26, 33.14, 31.00, 30.67, 25.69, 22.70. Found (%): C, 60.44; H, 5.31; N, 12.59; S, 14.78. Calc. for C_22_H_22_N_4_O_2_S (%): C, 60.25; H, 5.06; N, 12.78; S, 14.62.

#### 3.1.2. Synthesis of Compound **3** [61]

*4-{4-[2-(2-Aminoethyl)-4,5-dimethoxyphenyl]thiazol-2-yl}phenol hydrochloride* (**3**). The mixture of 1-(chloromethyl)-6,7-dimethoxy-3,4-dihydroisoquinoline (2.39 g, 0.01 мoль), 4-hydroxybenzothioamide (1.53 g, 0.01 мoль) and PrOH (15 mL) was boiled with stirring for 2 h. Then, it was cooled, and hydrochloride **3** was filtered off. Yield 2.65 г (67%), colorless crystals, m.p. 276–277 °C (PrOH). ^1^H NMR (400 MHz, DMSO-*d*_6_, δ, ppm): 3.15 (t, *J* 6.5, 2H, 2H-), 3.87 (s, 3H, OMe), 3.91 (s, 3H, OMe), 4.59 (t, *J* 6.5, 2H, 2H-), 7.04 (s, 1H, H-3″), 7.15 (d, *J* 8.4, 2H, H-3, H-5), 7.27 (s, 1H, H-6″), 7.44 (s, 1H, H-5′), 7.60–7.62 (m, 2H, NH_2_), 7.68 (d, *J* 8.6, 2H, H-2, H-6), 8.76 (s, 1H, OH), 10.94 (s, 1H, ^+^NH). ^13^C NMR (126 MHz, DMSO-*d*_6_, δ, ppm): 166.52, 158.49 (C-OH), 153.82, 152.46 (C-OMe), 151.30 (C-OMe), 135.15 (2C), 134.82, 134.65, 133.58 (2C), 117.55 (2C), 117.16, 114.29 (2C), 48.19 (2C, CH_3_), 43.62 (CH_2_NH), 32.15. Found (%): C, 58.26; H, 5.60; Cl, 9.32; N, 7.00; S, 8.34. Calc. for C_19_H_21_ClN_2_O_3_S (%): C, 58.08; H, 5.39; Cl, 9.02; N, 7.13; S, 8.16.

#### 3.1.3. General Procedure for the Synthesis of Compounds **4**–**7**

A mixture of 1-chloro-4-R-phthalazine (0.01 mol), 4-amino-N-(thiazol-2-yl) benzenesulfonamide (2.55 g, 0.01 mol) in methyl cellosolve (20 mL) was boiled for 30 min, cooled to 95–100 °C, and poured into 5% NH_4_OH (60 mL). Then, it was stirred for 1 h, filtered off, and washed with water (4–15 mL).

*4-[(4-Phenylphthalazin-1-yl)amino]-N-(thiazol-2-yl)benzenesulfonamide* (**4**). The starting compound was 1-chloro-4-phenylphthalazin. Yield 3.58 г (78%), colorless crystals, m.p. 297–299 °C (DMF). ^1^H NMR (400 MHz, DMSO-*d*_6_, δ, ppm): 6.70 (d, *J* 4.6, 1H, H-5′), 7.10 (d, *J* 4.6, 1H, H-4′), 7.52–7.60 (m, 3H, H-3. H-5, H-4″), 7.64–7. 67 (m, 2H, H-3″, H-5″), 7.76–7.81 (m, 2H, H-2, H-6), 7.86–8.01 (m, 3H, H-2″, H-6″, H-6′′′), 8.05–8.20 (m, 2H, H-5′′′, H-7′′′), 8.65 (d, *J* 8.2, 1H, H-8′′′), 9.47 (s, 1H, NH), 12.48 (s, 1H, NHSO_2_). ^13^C NMR (126 MHz, DMSO-*d*_6_, δ, ppm): 135.11 (2C), 132.97 (2C), 132.32, 130.15(3C), 129.19 (2C), 128.90 (4C), 127.17 (3C), 126.37, 119.89 (3C), 108.41. Found (%): C, 60.00; H, 3.51; N, 15.11; S, 14.21. Calc. for C_23_H_17_N_5_O_2_S_2_ (%): C, 60.11; H, 3.73; N, 15.24; S, 13.96.

*4-{[4-(4-Chlorophenyl)phthalazin-1-yl]amino}-N-(thiazol-2-yl)benzenesulfonamide* (**5**). The starting compound was 1-chloro-4-(4-chlorophenyl)phthalazine. Yield 4.30 г (87%), colorless crystals, m.p. 280–281 °C (DMF). ^1^H NMR (400 MHz, DMSO-*d*_6_, δ, ppm): 6.70 (d, *J* 4.6, 1H, H-5′), 7.10 (d, *J* 4.7, 1H, H-4′), 7.56–7.60 (m, 2H, H-3, H-5), 7.65–7.70 (m, 2H, H-3″, H-5″), 7.76–7.81 (m, 2H, H-2, H-6), 7.90–7.91 (m, 2H, H-6′′′, H-7′′′), 7.96–8.02 (m, 1H, H-5′′′,), 8.12–8.17 (m, 2H, H-2″, H-6″), 8.66 (d, *J* 8.2, 1H, H-8′′′), 9.49 (s, 1H, NH), 12.48 (s, 1H, NHSO_2_). ^13^C NMR (126 MHz, DMSO-*d*_6_, δ, ppm): 144.50 (NH-C), 135.80 (N=C), 134.08, 133.08, 132.43 (2C), 131.96 (3C), 128.98 (3C), 127.16 (3C), 126.18, 123.25, 119.99 (3C), 119.11, 108.43 (C=S). Found (%): C, 55.69; H, 3.05; Cl+S, 20.40; N, 14.02. Calc. for C_23_H_16_ClN_5_O_2_S_2_ (%): C, 55.92; H, 3.26; Cl, 7.18; N, 14.18; S, 12.98.

*4-[(4-Methylphthalazin-1-yl)amino]-N-(thiazol-2-yl)benzenesulfonamide* (**6**). The starting compound was 1-chloro-4-methylphthalazine. Yield 3.70 г (93%), colorless crystals, m.p. 284–286 °C (DMF). ^1^H NMR (400 MHz, DMSO-*d*_6_, δ, ppm): 2.91 (s, 3H, Me), 6.69–6.71 (m, 1H, H-5′), 7.08–7.10 (m, 1H, H-4′), 7.75–7.82 (m, 2H, H-3, H-5), 7.98–8.12 (m, 4H, H-2, H-6, H-6″, H-7″), 8.21–8.28 (m, 1H, H-5′′′), 8.79–8.85 (m, 1H, H-8′′′). ^13^C NMR (126 MHz, DMSO-*d*_6_, δ, ppm): 169.06 (N=CS), 152.73 (2C), 136.10, 134.24 (2C), 133.71, 127.55, 127.16 (3C), 126.87, 123.94, 120.75 (2C), 120.14, 108.50, 18.23 (CH_3_). Found (%): C, 54.16; H, 3.62; N, 17.44; S, 16.45. Calc. for C_18_H_15_N_5_O_2_S_2_ (%): C, 54.39; H, 3.80; N, 17.62; S, 16.13.

*4-{[4-(4-Methoxyphenyl)phthalazin-1-yl]amino}-N-(thiazol-2-yl)benzenesulfonamide* (**7**). The starting compound was 1-chloro-4-(4-methoxyphenyl)phthalazine. Yield 4.11 г (84%), colorless crystals, m.p. 142–143 °C (DMF). ^1^H NMR (400 MHz, DMSO-*d*_6_, δ, ppm): 3.89 (s, 3H, OMe), 6.69 (d, *J* 4.6, 1H, H-5′), 7.07–7.12 (m, 3H, H-4′, H-3″, H-5″), 7.56–7.63 (m, 2H, H-3, H-5), 7.76–7.79 (m, 2H, H-2″, H-6″), 7.85–7.90 (m, 1H, H-5′′′), 7.92–8.00 (m, 2H, H-6′′′, H-7′′′), 8.12–8.17 (m, 2H, H-2, H-6), 8.63 (d, *J* 8.2, 1H, H-8′′′), 9.42 (s, 1H, NH), 12.46 (s, 1H, NHSO_2_). ^13^C NMR (126 MHz, DMSO-*d*_6_, δ, ppm): 160.11 (4C), 132.85, 132.20, 131.47 (3C), 127.17 (2C), 126.49, 119.76 (3C), 114.34, 108.40, 55.69 (CH_3_). Found (%): C, 58.65; H, 3.70; N, 14.12; S, 13.37. Calc. for C_24_H_19_N_5_O_3_S_2_ (%): C, 58.88; H, 3.91; N, 14.31; S, 13.10.

N-[2-(4-Isopropylthiazol-2-yl)ethyl]-5-methyl-14-oxo-5a,6,12,14-tetrahydroindolo[2′,3′:4,5]pyrido[2,1-b]quinazoline-7(5H)-carboxamide (**8**). The starting compounds were 5-methyl-14-oxo-5a,6,12,14-tetrahydroindolo[2′,3′:4,5]pyrido[2,1-b]quinazoline-7(5H)-carboxylic acid and 2-(4-isopropylthiazol-2-yl)ethan-1-amine. Yield 3.54 g (71%), colorless crystals, m.p. 182–184 °C (EtOAc). ^1^H NMR (400 MHz, DMSO-d_6_, δ, ppm): 1.21 (s, 3H, Me), 1.24 (s, 3H, Me), 3.01–3.08 (m, 7H, NMe, 2H-α, 2H-7), 3.48 (d, J 6.8, 2H, 2H-α), 4.69–4.75 (m, 1H, CHMe_2_), 4.92 (d, J 2.6, 2H, 2H-13), 6.01 (s, 1H, H-6), 6.78 (s, 1H, H-5′), 7.08 (d, J 7.2, 1H, H-4), 7.15–7.24 (m, 4H, H-2, H-3, H-10, H-11), 7.35 (d, J 8.1, 1H, H-12), 7.48–7.57 (m, 2H, H-2, H-3), 7.94 (d, J 7.8, 1H, H-9), 8.08 (s, 1H, NH). ^13^C NMR (126 MHz, DMSO-d_6_, δ, ppm): 167.97 (CH_2_C=O), 166.83 (2C), 162.82 (N-C=O), 128.47 (3C), 122.72, 119.84 (2C), 112.85, 111.61 (3C), 110.54 (4C), 46.51 (2C), 39.12 (2C), 32.97 (3C), 30.62, 22.65 (2C, 2CH_3_), 20.16. Found (%): C, 67.11; H, 5.64; N, 14.00; S, 6.34. Calc. for C_28_H_29_N_5_O_2_S (%): C, 67.31; H, 5.85; N, 14.02; S, 6.42.

*4-Butyl-1-hydroxy-N-(6-methylbenzo[d]thiazol-2-yl)-3-oxo-3,4-dihydronaphthalene-2-carboxamide* (**9**). The starting compounds were 1-butyl-4-hydroxy-2-oxo-1,2-dihydroquinoline-3-carboxylic acid and 6-methylbenzo[d]thiazol-2-amine. Yield 3.67 g (69%), colorless crystals, m.p. 97–99 °C (CCl_4_). ^1^H NMR (400 MHz, DMSO-*d*_6_, δ, ppm): 1.05 (t, *J* 7.3, 3H, MeCH_2_), 1.46–1.59 (m, 2H, 2H-β), 1.69–1.79 (m, 2H, 2H-γ), 2.48–2.51 (m, 5H, Me-6′, DMSO), 2.82 (s, 1H, H-4), 4.33–4.38 (m, 2H, H-α), 7.22 (dd, *J* 8.3, 1.7, 1H, H-8), 7.37 (t, *J* 7.6, 1H, H-6), 7.56–7.70 (m, 3H, H-5, H-7, H-5′), 7.80–7.83 (m, 1H, H-7′), 8.21–8.24 (m, 1H, H-4′), 13.81 (s, 1H, NH), 15.19 (s, 1H, OH). ^13^C NMR (126 MHz, DMSO-*d*_6_, δ, ppm): 170.85 (C=O), 166.82 (2C), 160.01, 151.12, 140.11, 135.47, 131.56, 129.78, 123.47 (2C), 120.05 (2C), 109.13 (2C), 108.87, 99.95, 52.61 (CH_2_N), 29.84, 20.15, 19.23, 11.45 (CH_3_).

### 3.2. Biological Evaluation

#### 3.2.1. Antibacterial Action 

The following Gram-negative bacteria, *Escherichia coli* (ATCC 35210), *Enterobacter cloacae (clinical isolate), Salmonella Typhimurium* (ATCC 13311), as well as Gram-positive bacteria, *Listeria monocytogenes* (NCTC 7973), *Bacillus cereus* (clinical isolate), and *Staphylococcus aureus* (ATCC 6538), were used. The bacterial strains are deposited at Mycological Laboratory, Department of Plant Physiology, Institute for Biological Research “Siniša Stankovic”—National Institute of Republic of Serbia, Belgrade, Serbia.

The minimum inhibitory and bactericidal (MIC/MBC) concentrations were defined as described previously [62,63]. Resistant strains used were isolates of *S. aureus*, *E. coli*, and *P.aeruginosa*, obtained as reported in Kartsev et al. [63].

#### 3.2.2. Antifungal Activity

The examined strains were: Aspergillus niger (ATCC 6275), Aspergillus fumigatus (ATCC 1022), Aspergillus versicolor (ATCC 11730), Penicillium funiculosum (ATCC 36839), Trichoderma viride (IAM 5061), and Penicillium verrucosum var. cyclopium (food isolate). All experiments were performed in triplicate [64,65]. 

#### 3.2.3. Inhibition of Biofilm Formation

The assays were performed as described before [66,67]. Briefly, *P. aeruginosa* resistant strain was incubated with MIC and subMIC of the tested compounds in tryptic soy broth enriched with 2% glucose at 37 °C for 24 h. Afterwards, each well was washed twice with sterile Phosphate buffered saline, pH 7.4 (PBS), and fixed with methanol for 10 min. Methanol was then removed, and the plate was air-dried. The biofilm was stained with 0.1% crystal violet (Bio-Merieux, France) for 30 min. The wells were washed with water, air-dried, and color dissolved in 96% ethanol (Zorka, Serbia). The absorbance was measured at 620 nm on a Multiskan FC Microplate Photometer, Thermo Scientific. The percentage of inhibition of biofilm formation was calculated by the formula:

[(A620control − A620sample)/A620control] × 100.(1)

### 3.3. Molecular Modeling Studies

The ligand preparation done by using chemdraw12.0, and geometries were optimized using LigandScout 4.4.5. The “Build/check/repair model” for the session “Prepare PDB file for docking programs” module was used for proteins preparation. For the final preparation of both ligands and protein preparation, Wizard of AutoDock tools 1.5.6 is used. Autodock 4 (ver. 4.2.6) was employed for docking simulations and Autogrid4 for affinity grid maps preparation. The resulting poses and potential interactions were visualized using LigandScout 4.4.5.

X-ray crystal structures of *E. coli* DNA GyrB, thymidylate kinase, *E. coli* MurA, *E. coli* primase, *E. coli* MurB, DNA topoisomerase IV, and CYP51 of *C*. *albicans* (PDB ID: 1KZN, AQGG, 1DDE, JV4T, 2Q85, 1S16, and 5V5Z, respectively) with bound inhibitors were retrieved from Brookhaven Protein Data Bank (PDB). The pdb files of proteins were submitted to “Build/check/repair model” for the session “Prepare PDB file for docking programs”; missing side chains were modeled in, water positions and symmetry were corrected, and hydrogen atoms were added. Only chain A of each enzyme of the repaired pdb file was evaluated and passed to AutodockTools (ADT ver.1.5.6) for PDBQT file preparation. ADT assigned polar hydrogens, water molecules and nonstandard residues were removed, so only polar hydrogen was maintained, and Gasteiger charges were computed for protein atoms. AutoDock saved the prepared file in PDBQT format. 

All molecules were sketched in Chemdraw12.0 program. The geometry of built compounds was optimized using the molecular mechanical force fields 94 (MMFF94) energy via LigandScout [68], partial charges were also calculated, comformers of each ligand were generated, and the one with the best conformation was maintained and saved as mol2 file that was passed, as usual, to ADT for PDBQT file preparation. There, polar hydrogen was added to each structure, followed by computing Gasteiger and Kollman charges and the torsions.

Autodock 4 (ver. 4.2.6) was employed for docking simulations. A computationally (relatively) ‘hybrid’ force field that contains terms based on molecular, mechanics, and empirical terms is used by AutoDock. The evaluation step includes: First, calculation of the energy of protein and ligand in the unbound state. Second, calculation of the energy of the ligand–protein complex. Third, taking the difference between first and second steps.

$$\Delta G=\left(V_{\mathrm{bound}}^{L-L}-V_{\mathrm{unbound}}^{L-L}\right)+\left(V_{\mathrm{bound}}^{P-P}-V_{\mathrm{unbound}}^{P-P}\right)+\left(V_{\mathrm{bound}}^{P-L}-V_{\mathrm{unbound}}^{P-L}+\Delta S_{\mathrm{conf}}\right)$$

where *P* refers to the protein, *L* refers to the ligand, *V* are the pair-wise evaluations mentioned above, and Δ*S*_conf_ denotes the loss of conformational entropy upon binding [69]. The ligand molecule is in an arbitrary conformation, orientation, and position, and this molecular docking program finds favorable poses in a protein-binding site using Lamarckian genetic algorithms implemented therein to search for the best conformers.

A Lamarckian genetic algorithm was used as the search engine, with a total of 100 runs. The region of interest, used by Autodock4 for docking runs and by Autogrid4 for affinity grid maps preparation, was defined in such a way to comprise the whole catalytic binding site using a grid of 50 × 50 × 50 points with a grid space of 0.375 Å. All parameters used in docking were default. The translation, quaternion, and torsions steps were taken from default values in AutoDock. The Lamarckian genetic algorithm and the pseudo-Solis and Wets methods were applied for minimization using default parameters. The number of docking runs was 100. After docking, the 100 solutions were clustered into groups, with RMS lower than 1.0 Ε. The clusters were ranked by the lowest energy representative of each cluster. Upon completion of docking, the best poses were screened by examination of binding energy (Δ*G*_binding_, kcal/mol) and number in cluster. In order to describe the ligand-binding pocket interactions, the top-ranked binding mode found by AutoDock in complex with the binding pocket of enzyme was selected. The resulting poses and potential interactions were visualized using LigandScout.

## 4. Conclusions

In this work, three structural series of new thiazole derivatives were synthesized and evaluated for their antibacterial and antifungal activity against a series of bacterial and fungal pathogens. The antibacterial activity of the tested compounds is moderate to good, with MIC at 0.23–>3.75 mg/mL and MBC at 0.35–>3.75 mg/mL. Compounds **4** and **9** demonstrated the best activity among the tested compounds against *E. coli* and *B. cereus* and *S.Typhimurium*, respectively, with MIC/MBC at 0.17/0.23 mg/mL, respectively.

Three of the most active compounds (**2**, **3**, and **4**) were also evaluated against three resistant strains, *MRSA, E. coli*, and *P. artuginosa*, demonstrating better activity than the reference drugs against MRSA, while compound **4** also was active against *P. aeruginosa.*

According to the results on antifungal activity, all compounds are active, but the best activity was observed for compound **8**, with MIC and MFC in the range of 0.08–0.23 and 0.11–0.47 mg/mL, respectively.

Docking analysis indicated a probable involvement of MurB inhibition in the antibacterial mechanism of the compounds tested, while the docking analysis to 14α-lanosterol demethylase (CYP51) of *Candida albicans* indicated a probable implication of CYP51 reductase at the antifungal activity of the compounds. Finally, compound **8** showed the best drug-likeness model score.

## Figures and Tables

**Table 1:** Antibacterial activity of the title compounds (MIC/MBC in mg/mL).

№	Compounds		*S.a.*	*B.c.*	*L.m.*	*E.c.*	*S.T.*	*En.cl.*
**1**	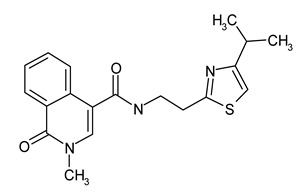	MIC	0.70 ± 0.19	0.35 ± 0.09	0.35 ± 0.09	>3.75	>3.75	0.23 ± 0.00
		MBC	0.94 ± 0.00	0.47 ± 0.00	0.47 ± 0.00	>3.75	>3.75	0.47 ± 0.00
**2**	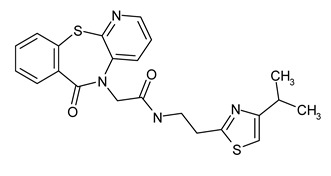	MIC	0.94 ± 0.00	0.47 ± 0.00	0.70 ± 0.19	0.35 ± 0.09	0.35 ± 0.09	0.35 ± 0.09
		MBC	1.88 ± 0.00	0.94 ± 0.00	0.94 ± 0.00	0.47 ± 0.00	0.47 ± 0.00	0.47 ± 0.00
**3**	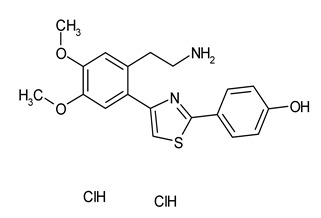	MIC	0.70 ± 0.19	0.23 ± 0.00	0.70 ± 0.19	0.23 ± 0.00	0.23 ± 0.00	0.70 ± 0.19
		MBC	0.94 ± 0.00	0.47 ± 0.00	0.94 ± 0.00	0.47 ± 0.00	0.47 ± 0.00	0.94 ± 0.00
**4**	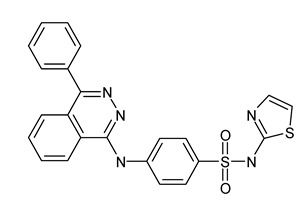	MIC	1.41 ± 0.38	0.70 ± 0.19	0.70 ± 0.19	0.17 ± 0.00	0.70 ± 0.19	0.70 ± 0.19
		MBC	1.88 ± 0.00	0.94 ± 0.00	0.94 ± 0.00	0.23 ± 0.00	0.94 ± 0.00	0.94 ± 0.00
**5**	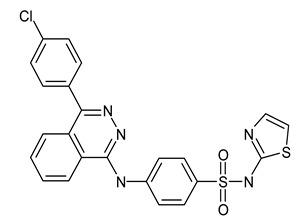	MIC	1.41 ± 0.38	0.70 ± 0.19	0.70 ± 0.19	0.23 ± 0.00	0.47 ± 0.00	0.70 ± 0.19
		MBC	1.88 ± 0.00	0.94 ± 0.00	0.94 ± 0.00	0.47 ± 0.00	0.94 ± 0.00	0.94 ± 0.00
**6**	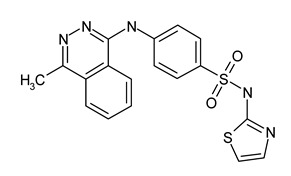	MIC	2.31 ± 0.76	1.41 ± 0.38	0.70 ± 0.19	>3.75	0.70 ± 0.19	0.70 ± 0.19
		MBC	3.75 ± 0.00	1.88 ± 0.00	0.94 ± 0.00	>3.75	0.94 ± 0.00	0.94 ± 0.00
**7**	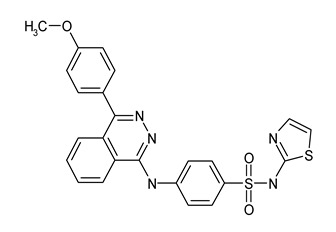	MIC	1.41 ± 0.00	0.70 ± 0.19	0.70 ± 0.19	0.35 ± 0.09	0.70 ± 0.19	0.70 ± 0.19
		MBC	1.88 ± 0.00	0.94 ± 0.00	0.94 ± 0.00	0.47 ± 0.00	0.94 ± 0.00	0.94 ± 0.00
**8**	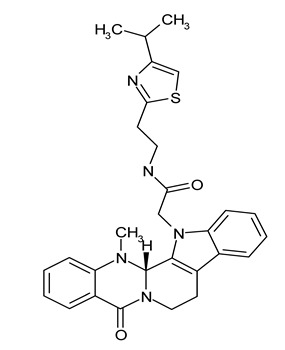	MIC	1.41 ± 0.38	0.47 ± 0.00	0.35 ± 0.09	>3.75	0.35 ± 0.09	0.23 ± 0.00
		MBC	1.88 ± 0.00	0.94 ± 0.00	0.47 ± 0.00	>3.75	0.47 ± 0.00	0.47 ± 0.00
**9**	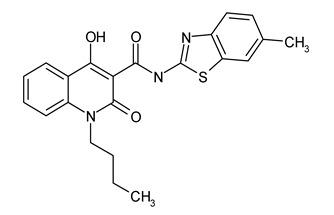	MIC	0.94 ± 0.00	0.17 ± 0.00	0.35 ± 0.09	0.70 ± 0.19	0.17 ± 0.19	0.70 ± 0.19
		MBC	1.88 ± 0.00	0.23 ± 0.00	0.47 ± 0.00	0.94 ± 0.00	0.23 ± 0.00	0.94 ± 0.00
**Streptomycin**		MIC	0.10 ± 0.00	0.02 ± 0.00	0.15 ± 0.00	0.10 ± 0.00	0.10 ± 0.00	0.02 ± 0.00
		MBC	0.20 ± 0.01	0.05 ± 0.00	0.30 ± 0.01	0.20 ± 0.00	0.20 ± 0.01	0.05 ± 0.00
**Ampicillin**		MIC	0.10 ± 0.00	0.10 ± 0.00	0.15 ± 0.00	0.15 ± 0.00	0.10 ± 0.00	0.10 ± 0.00
		MBC	0.15 ± 0.00	0.15 ± 0.00	0.30 ± 0.02	0.20 ± 0.01	0.20 ± 0.00	0.15 ± 0.01

**Table 2:** Antibacterial activity and nhibition of biofil formation against resistant strains (MIC/MBC in mg/mL).

Compound		*MRSA*	*P.a.*	*E.c.*	MIC	0% MIC
**2**	MIC	0.94 ± 0.00	0.23 ± 0.00	0.94 ± 0.00	14.59	7.08
	MBC	1.88 ± 0.00	0.47 ± 0.00	1.88 ± 0.00		
**3**	MIC	0.47 ± 0.00	0.23 ± 0.00	0.47 ± 0.00	19.97	8.84
	MBC	0.94 ± 0.00	0.47 ± 0.00	0.94 ± 0.00		
**4**	MIC	0.94 ± 0.00	0.23 ± 0.00	0.94 ± 0.00	4.31	NE
	MBC	1.88 ± 0.00	0.47 ± 0.00	1.88 ± 0.00		
**Streptomycin**	MIC	0.10 ± 0.00	0.05 ± 0.00	0.10 ± 0.00	71.94	55.42
	MBC	/	0.10 ± 0.00	0.20 ± 0.00		
**Ampicillin**	MIC	/	0.20 ± 0.01	0.20 ± 0.00	67.36	30.35
	MBC	/	/	/		

**Table 3:** Antifungal activity of thiazole derivatives. (MIC and MBC in mg/mL).

Compounds		*A.f.*	*A.n.*	*A.v.*	*P.f.*	*T.v.*	*P.v.c.*
**1**	MIC	0.35 ± 0.08	0.08 ± 0.00	0.23 ± 0.00	0.17 ± 0.00	0.11 ± 0.00	0.17 ± 0.00
	MFC	0.47 ± 0.00	0.11 ± 0.00	0.47 ± 0.00	0.23 ± 0.00	0.23 ± 0.00	0.23 ± 0.00
**2**	MIC	0.35 ± 0.08	0.23 ± 0.00	0.35 ± 0.08	0.23 ± 0.00	0.17 ± 0.00	0.35 ± 0.08
	MFC	0.47 ± 0.00	0.47 ± 0.00	0.47 ± 0.00	0.47 ± 0.00	0.23 ± 0.00	0.47 ± 0.00
**3**	MIC	0.17 ± 0.00	0.11 ± 0.00	0.23 ± 0.00	0.23 ± 0.00	0.11 ± 0.00	0.35 ± 0.08
	MFC	0.23 ± 0.00	0.23 ± 0.00	0.47 ± 0.00	0.47 ± 0.00	0.23 ± 0.00	0.47 ± 0.00
**4**	MIC	0.47 ± 0.00	0.47 ± 0.00	0.23 ± 0.00	0.35 ± 0.08	0.23 ± 0.00	0.23 ± 0.00
	MFC	0.94 ± 0.00	0.94 ± 0.00	0.47 ± 0.00	0.47 ± 0.00	0.47 ± 0.00	0.47 ± 0.00
**5**	MIC	0.23 ± 0.00	0.06 ± 0.00	0.23 ± 0.00	0.23 ± 0.00	0.17 ± 0.00	0.17 ± 0.00
	MFC	0.47 ± 0.00	0.11 ± 0.00	0.47 ± 0.00	0.47 ± 0.00	0.23 ± 0.00	0.23 ± 0.00
**6**	MIC	0.47 ± 0.00	0.23 ± 0.00	0.47 ± 0.00	0.23 ± 0.00	0.17 ± 0.00	0.47 ± 0.00
	MFC	0.94 ± 0.00	0.47 ± 0.00	0.94 ± 0.00	0.47 ± 0.00	0.23 ± 0.00	0.94 ± 0.00
**7**	MIC	0.47 ± 0.00	0.23 ± 0.00	0.23 ± 0.00	0.47 ± 0.00	0.47 ± 0.00	0.23 ± 0.00
	MFC	0.94 ± 0.00	0.47 ± 0.00	0.47 ± 0.00	0.94 ± 0.00	0.94 ± 0.00	0.47 ± 0.00
**8**	MIC	0.23 ± 0.00	0.11 ± 0.00	0.11 ± 0.00	0.17 ± 0.00	0.08 ± 0.00	0.11 ± 0.00
	MFC	0.47 ± 0.00	0.23 ± 0.00	0.23 ± 0.00	0.23 ± 0.00	0.11 ± 0.00	0.23 ± 0.00
**9**	MIC	0.23 ± 0.00	0.17 ± 0.00	0.17 ± 0.00	0.17 ± 0.00	0.06 ± 0.00	0.17 ± 0.00
	MFC	0.47 ± 0.00	0.23 ± 0.00	0.23 ± 0.00	0.23 ± 0.00	0.11 ± 0.00	0.23 ± 0.00
Bifonazole	MIC	0.15 ± 0.00	0.15 ± 0.00	0.10 ± 0.00	0.20 ± 0.00	0.15 ± 0.00	0.10 ± 0.00
	MFC	0.20 ± 0.00	0.20 ± 0.00	0.20 ± 0.00	0.25 ± 0.00	0.20 ± 0.00	0.20 ± 0.00
Ketoconazole	MIC	0.20 ± 0.00	0.20 ± 0.00	0.20 ± 0.00	0.20 ± 0.00	1.00 ± 0.01	0.20 ± 0.00
	MFC	0.50 ± 0.00	0.50 ± 0.00	0.50 ± 0.00	0.50 ± 0.00	1.50 ± 0.00	0.30 ± 0.010

**Table 4:** Molecular docking free binding energies (kcal/mol) to antibacterial targets.

Comp.	Est. Binding Energy (kcal/mol)					I-H *E. coli* MurB	Residues *E. coli* MurB
	*E. coli* Gyrase 1KZN	Thymidylate Kinase 4QGG	*E. coli* Primase 1DDE	*E. coli* MurA JV4T	*E. coli* MurB 2Q85		
**1**	−3.46	-	-	−3.85	−7.02	1	Arg158
**2**	−4.52	−3.18	−2.94	−5.03	−9.16	1	Ser229
**3**	−4.88	−2.71	-	−5.14	−9.96	2	Gly47, Ser229
**4**	−3.82	−3.11	-	−4.69	−8.70	1	Ser229
**5**	−3.96	-	−2.91	−3.67	−7.53	1	Arg213
**6**	−4.62	−2.54	−3.47	−5.75	−8.65	2	Gly122, Arg213
**7**	−2.66	-	-	−4.12	−7.05	1	Arg213
**8**	−2.15	−3.54	−1.28	−4.63	−8.51	2	Arg213, Ser229
**9**	−2.74	-	-	−3.79	−8.52	1	Ser229

**Table 5:** Molecular docking free binding energies (kcal/mol) to antifungal targets.

	Est. Binding Energy (kcal/mol)				
N/N	DNA TopoIV 1S16	CYP51 of *C. albicans* *5V5Z*	I-H	Residues CYP51 of *C. albicans*	Interactions with HEM601
**1**	−2.17	−9.15	1	Tyr132	Hydrophobic, Aromatic
**2**	−3.10	−7.95	1	Tyr132	Hydrophobic
**3**	-	−8.11	1	Tyr118	Hydrophobic
**4**	-	−7.52	1	Tyr118	Hydrophobic
**5**	−1.42	−7.50	1	Tyr118	Hydrophobic
**6**	−1.56	−8.64	1	Tyr64	Hydrophobic
**7**	−2.25	−7.12	-	-	Hydrophobic, Aromatic
**8**	-	−7.03	-	-	Hydrophobic
**9**	−2.71	−9.21	1	Tyr132	Hydrophobic, Aromatic
**Ketoconazole**	-	-8.23	1	Tyr64	Hydrophobic, Aromatic

**Table 6:** Drug-likeness predictions of tested compounds.

No	MW	Number of HBA ^a^	Number of HBD ^b^	Log *P*_o/w_ (iLOGP) ^c^	Log S ^d^	TPSA ^e^	BBB Permeant ^f^	Lipinski, Ghose, Veber, Egan, and Muegge Violations	Bioavailability Score	Drug-Likeness Model Score
**1**	355.45	3	1	2.8	Poorly soluble	92.23	No	0	0.55	1.03
**2**	438.12	4	1	3.37	Poorly soluble	128.73	No	0	0.55	1.09
**3**	339.16	5	3	2.33	Poorly soluble	93.39	No	0	0.55	0.01
**4**	459.54	5	2	3.00	Poorly soluble	133.49	No	0	0.55	−0.12
**5**	493.99	5	2	3.20	Poorly soluble	133.49	No	3 *	0.55	−0.13
**6**	396.49	5	1	2.26	Poorly soluble	121.46	No	0	0.55	0.27
**7**	489.57	6	2	3.07	Poorly soluble	142.72	No	3 *	0.55	0.27
**8**	513.65	3	1	3.73	Poorly soluble	98.71	No	1 **	0.55	1.37
**9**	407.49	4	2	2.86	Poorly soluble	112.46	No	0	0.55	0.43

^a^ Number of hydrogen bond acceptors; ^b^ number of hydrogen bond donors; ^c^ lipophilicity; ^d^ water solubility (SILICOS-IT (S = Soluble)); ^e^ topological polar surface area (Å^2^); ^f^ blood–brain barrier permeant; * Ghose: 3 violations: MW > 480, WLOGP > 5.6, MR > 130; ** Lipinski: 1 violation: MW > 500; and Ghose: 2 violations: MW > 480, MR > 130.

## Data Availability

Not applicable.
